# Dexmedetomidine infusion as an analgesic adjuvant during laparoscopic сholecystectomy: a randomized controlled study

**DOI:** 10.1186/s12871-018-0508-6

**Published:** 2018-04-20

**Authors:** Kateryna Bielka, Iurii Kuchyn, Volodymyr Babych, Kseniia Martycshenko, Oleksii Inozemtsev

**Affiliations:** Department of Surgery, Anesthesiology and Intensive Care, Postgraduate Institute of Bogomolets National Medical University, 36 Peremohy avenue, Kiev, 03055 Ukraine

**Keywords:** Postoperative pain, Laparoscopic cholecystectomy, Dexmedetomidine, Randomized controlled trial

## Abstract

**Background:**

Dexmedetomidine (DEX) has sedative, sympatholytic and analgesic effects and might be beneficial if used as an adjuvant to: improve analgesia; modulate haemodynamic responses to intubation and pneumoperitoneum and; reduce the number of opioid-associated adverse events. The aim of this study was to evaluate the efficacy and safety of DEX infusion during elective laparoscopic cholecystectomy (LC).

**Methods:**

A randomized, single-centre, parallel-group, placebo-controlled study was carried out between May 2016 and June 2017. Adult patients (18–79 years) with American Society of Anesthesiology (ASA) physical status I–II were randomly assigned to 0.5 μg/kg/h DEX infusion from induction of anaesthesia to extubation (Group D; *n* = 30) or normal saline infusion (Group C; n = 30). The primary efficacy outcomes were postoperative morphine consumption. Secondary efficacy outcomes included: time to first use of rescue analgesia; postoperative morphine consumption; intraoperative fentanyl consumption; time from end of surgery to extubation; lengths of intensive care unit (ICU) and general ward stay; degree of postoperative pain 3, 6, 12 and 24 h after surgery; incidence of persistent post-surgical pain.

**Results:**

DEX infusion was associated with a decrease in postoperative morphine consumption (*p* = 0.001), lower incidence of severe postoperative pain (odds ratio [OR] 9, 95% confidence interval [CI] 1.1–77, *p* = 0.04) and significantly longer time to first use of rescue analgesia (*p* = 0.001). Group D also had significantly lower fentanyl consumption both intraoperatively (p = 0.001) and in the time from end of surgery to extubation (p = 0.001) plus decreased incidence of persistent post-surgical pain (OR 14.5, 95% CI 1.7–122, *p* = 0.005). The incidence of postoperative nausea and vomiting was lower in Group D than Group C (OR 5, 95% CI 1.1–26, p = 0.005). Median pain intensity did not differ between the groups 3, 6, 12 or 24 h after surgery and there were no inter-group differences in the lengths of ICU stay or overall hospital stay between groups. The incidence of hypertension was significantly higher in Group C (OR 13.8, 95% CI 4–48, *p* < 0.0001); there were no inter-group differences in incidences of hypotension and bradycardia.

**Conclusions:**

Intraoperative DEX infusion is safe and effective for improving analgesia during and after elective LC. DEX appears to significantly reduce the number of patients with severe postoperative pain, postoperative morphine consumption and prolong time to first use of rescue analgesia.

**Trial registration:**

ClinicalTrials.gov: Retrospectively registered on July 7, 2017, NCT03211871.

## Background

Laparoscopic cholecystectomy (LC) is usually associated with less pain compared with an open approach. Postoperative pain is nevertheless still the main complaint after LC and one which often prolongs hospital stay [1]. Moderate abdominal and shoulder pain is experienced by 36–63% of patients 24–48 h after LC [2] and up to 13% of patients experience severe pain [3].

Opioids remain one of the main options for postoperative pain relief after LC and are widely used [3, 4]. However, opioids have side effects such as sedation, respiratory depression, nausea and vomiting, paralytic ileus and urinary retention which may outweigh the benefits of analgesia, especially after abdominal surgery. It has been recommended to use opioids after abdominal surgery only when non-opioid drugs provide insufficient analgesia [1]. There is thus a need to study and evaluate newer non-opioid pain medications after LC as part of an opioid-reduction strategy.

Dexmedetomidine (DEX) is a centrally-acting alpha-2-adrenoceptor agonist that has sedative, sympatholytic and analgesic effects. This pharmacodynamic profile identifies DEX as a potential non-opioid adjuvant to improve analgesia and haemodynamic responses to intubation and pneumoperitoneum and to decrease the number of opioid-associated adverse events. The aim of this study was to evaluate the efficacy and safety of DEX infusion during elective LC. The primary efficacy outcome was: number of patients with severe pain. Secondary efficacy outcomes included: time to first use of rescue analgesia; postoperative morphine consumption; intraoperative fentanyl consumption; time from end of surgery to extubation; lengths of intensive care unit (ICU) and general ward stay; degree of postoperative pain 3, 6, 12 and 24 h after surgery; incidence of persistent post-surgical pain.

## Methods

A randomized, single-centre, controlled study was carried out between May 2016 and June 2017 at the Department of Surgery, Anesthesiology and Intensive Care of the Postgraduate Institute of Bogomolets National Medical University, Kiev, Ukraine. The study design was approved by the Ethical Committee at Bogomolets National Medical University.

Sixty patients who elected to undergo LC were included in the study. Inclusion criteria comprised age 18–79 years and American Society of Anesthesiology (ASA) physical status I–II. Exclusion criteria were pregnancy or lactation, severe systemic disease (ASA physical status III) and use of beta-blockers or calcium-channel blockers.

After the primary patient assessment, eligible participants were assigned in a 1:1 ratio to either the intervention (Group D) or control (Group C) groups using random assignment in blocks of four. The randomization sequence was generated using a computer algorithm. Randomization and data analysis were conducted by an independent blinded member of the research team, so the PROBE design was used [5].

Group D received a 0.5 μg/kg/h DEX infusion from induction of anaesthesia to extubation and Group C received a normal saline infusion. To prepare the infusion, 2 ml of DEX containing 100 μg of the drug was diluted to 50 ml with normal saline, resulting in a final concentration of 4 μg/kg. DEX or normal saline infusion was given via a BBraun Space infusion pump.

After transferring the patient to the operation room, a Philips vital signs monitor and bispectral index (BIS) and analgesia nociception index (ANI) monitors were attached to determine pulse, heart rate, electrocardiogram (ECG), arterial pressure (AP) and oxygen saturation. A peripheral intravenous cannula was inserted for intravenous (i.v.) fluids and an infusion pump (separate line). Patients did not receive premedication. Before induction they received 50 mg of i.v. dexketoprofen and 40 mg of omeprazole.

Pre-oxygenation was performed for 2 min before induction of anaesthesia with 2 mg/kg i.v. propofol and 1.5 mg/kg i.v. succinylcholine. After intubation, anaesthesia was maintained with sevoflurane and atracurium bromide. Patients were ventilated using a circle system with a target CO_2_ level of 35–45 mmHg. The BIS monitor target was 40–60 and the ANI monitor target was 50–70. Anaesthetics and drug infusions were stopped at the end of surgery.

The primary efficacy outcome was: postoperative morphine consumption during first 24 h and cumulative during hospital stay. Subcutaneous injection of 5 mg morphine hydrochloride was used as a rescue analgesic, patient control analgesia (PCA) was not used in this study.

Secondary efficacy outcomes included:

- time to first use of rescue analgesia
- number of patients with severe pain
- intraoperative fentanyl consumption
- time from end of surgery to extubation
- lengths of intensive care unit (ICU) and general ward stay
- degree of postoperative pain 3, 6, 12 and 24 h after surgery
- incidence of persistent post-surgical pain (6 month after surgery).

Time to first use of rescue analgesia was estimated as the time from the end of anaesthesia to the time postoperatively when the patient requested analgesia or had a VRS score of ≥4. Severe pain was estimated using a verbal rating scale (VRS) [6] and defined as a score of ≥7 for ≥30% of the time during the first 48 h after surgery. Non-steroidal anti-inflammatory drugs (150 mg/day dexketoprofen) and paracetamol (3 g/day) were prescribed routinely.

During the first 48 h after surgery, patients in both groups were evaluated by the nursing staff using the Richmond Agitation-Sedation Scale (RASS) for sedation (every 2 h) and the VRS (on a scale ranging from zero to 10, every 2 h for or prior to rescue analgesia).

Safety was assessed by monitoring vital signs and recording adverse events. During anaesthesia, all patients underwent continuous ECG, BIS, pulse oximetry and capnographic monitoring. AP was measured every 3–5 min. An adverse event was recorded if systolic blood pressure was < 90 or > 160 mmHg or if heart rate was < 50 or > 110 beats/min. Interventions for bradycardia, tachycardia, hypertension and hypotension comprised titration or interruption of study agent or additional drug therapy. Postoperative sedation was recorded if the patient had a RASS score of ≤ − 3 during the 24-h period after surgery.

Persistent post-surgical pain was evaluated 6 month after surgery using the following criteria [7]: the pain should be of at least 2 months’ duration; other causes for the pain should be excluded; and the possibility that the pain is a continuation of a pre-existing problem should be explored and exclusion attempted.

Sample size was calculated using MedCalc Software version 16.8.4 (MedCalc Software bvba, Acacialaan 22, 8400 Ostend, Belgium). Based on minimum mean difference of 25% in morphine consumption [8, 9] with α = 0.01 and β = 0.20, sample size for each group was estimated as 18. Rounding up this figure, we took 30 patients in each group. Statistical analysis was performed using Statistica 8.0 and R software (StatSoft Inc., Tulsa, OK, USA). Categorical data are presented as proportions and continuous data as medians with 25–75% interquartile ranges (IQRs). Chi-squared testing demonstrated that all of the study variables were discrete. To assess significance levels, a two-tailed Mann–Whitney U-test and Fisher’s exact test were used. A *p*-value of < 0.05 was considered significant.

## Results

**Fig. 1:**
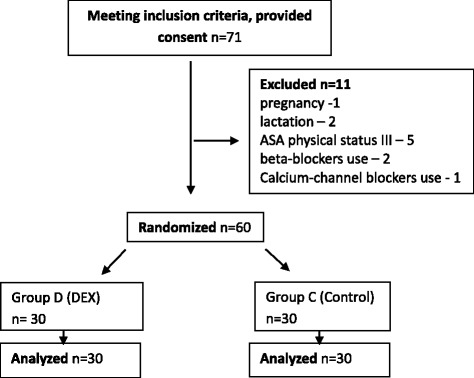
CONSORT flowchart

A total of 60 patients were randomized (30 in each group). There were no significant differences between the study groups regarding demographic characteristics, comorbidities or ASA physical status (Table 1 and Fig. 1).

**Table 1:** Demographic data and comorbidities

Characteristic	Group D	Group C	*p*-value
Females; n (%)	27 (90)	26 (87)	0.3
Age (years)	55 (49–61)	53 (49–66)	0.5
Comorbidities			
Arterial hypertension; n (%)	7/30 (23)	9/30 (30)	0.2
Diabetes mellitus; n (%)	3/30 (10)	2/30 (7)	0.3
Chronic obstructive lung disease; n (%)	2/30 (7)	1/30 (4)	0.4
ASA physical status	2 (1–2)	2 (1–2)	1
Overweight (WHO critera); n (%)	2 (7)	2 (7)	1
Obesity (WHO criteria); n (%)	2 (7)	2 (7)	1

Unless specified otherwise, values are expressed as medians, with 25–75% interquartile ranges in parentheses. ASA: American Society of Anaesthesiology

The main outcomes of the study are presented in Tables 2 and 3. As shown in Table 2, intraoperative DEX infusion favourably influenced many of the primary and secondary outcomes, including opioid consumption, incidence of severe postoperative pain and opioid-related adverse events. No significant effect was demonstrated on postoperative pain level. DEX infusion was associated with a lower incidence of severe postoperative pain (OR 9, 95% CI 1.1–77, *p* = 0.04), a significantly longer time to first use of rescue analgesia (*p* = 0.001) and a marked decrease in postoperative morphine consumption (median 5 mg/24 h vs. 15/24 h in Group C; p = 0.001). There were also no differences in the lengths of ICU stay or hospital stay between Groups D and C: 14 h (IQR 12–21 h) and 13 h (IQR 12–20 h), *p* = 0.9 and 72 h (IQR 70–80 h) and 74 h (IQR 72–82 h), *p* = 0.8, respectively. Also patients in Group D also had significantly lower intraoperative fentanyl consumption (p = 0.001), a shorter time from end of surgery to extubation (p = 0.001) and a decreased incidence of persistent post-surgical pain (OR 14.5, CI 95% 1.7–122, *p* = 0.005).

**Table 2:** Efficacy outcomes

Outcome	Group D	Group C	*p*-value
Postoperative morphine consumption in 24 h (mg)	5 (0–10)	15 (10–20)	0.001^2^
Cumulative morphine consumption (mg)	15 (10–25)	30 (20–30)	0.001^2^
Severe pain incidence; n (%)	1 (3)	7 (23)	0.04^1^
Time to first use of rescue analgesia (min)	180 (130–210)	80 (60–120)	0.001^2^
Time to extubation (min)	10 (5–10)	20 (15–20)	0.001^2^
Postoperative pain level (VRS score)			
3 h	3 (3–4)	4 (4–5)	0.067^2^
6 h	4 (4–5)	5 (4–5)	0.08^2^
12 h	3 (3–3)	4 (4–5)	0.33^2^
24 h	4 (4–4)	4 (4–4)	0.72^2^
Intraoperative fentanyl consumption (mg)	0.5 (0.4–0.6)	0.6 (0.5–0.7)	0.03^2^
Persistent postsurgical pain incidence; n (%)	1 (3)	10 (33)	0.005^1^

Unless specified otherwise, values are expressed as medians, with 25–75% interquartile ranges in parentheses. VRS: verbal rating scale

^1^Fisher test, ^2^Mann-Whitney test

**Table 3:** Adverse events rates

Adverse event	Group D	Group C	OR (95% CI)	*p*-value (Fisher test)
Hypotension	8 (27)	4 (13)	2.2 (0.6–8)	0.25
Hypertension	5 (15)	22 (33)	13.8 (4–48)	< 0.001
Tachycardia	1 (3)	9 (30)	12.4 (1.5–106)	0.02
Bradycardia	6 (20)	2 (7)	3.5 (0.6–19)	0.15
Postoperative sedation	6 (15)	5 (20)	1.25 (0.3–5)	0.7
Nausea/vomiting	2 (6)	8 (27)	5 (1.1–26)	0.05
Pruritus	0 (0)	2 (6)	5 (0.2–116)	0.28

Unless specified otherwise, values are expressed as numbers of patients, with percentages in parentheses. OR: odds ratio; CI: confidence interval

Patients randomized to DEX were significantly less likely to experience post-operative nausea or vomiting or to develop hypertension or tachycardia (Table 3). Few patients in both groups had postoperative sedation (RASS -1 to − 2), which resolve within 2–3 h. No severe complications or side effects were noted; all patients in both groups were discharged from hospital.

## Discussion

Uncontrolled postoperative pain is associated with increased morbidity and complications, prolonged hospital stays, and the risk of chronic postsurgical pain [10]. Opioids are mostly used for postopoperative analgesia, even after minimally invasive and laparoscopic surgery, although they have significant side effects (nausea, ileus, inspiratory depression, delay in mobilisation and rehabilitation). In the last decades non-opioid analgesic strategies become more important, to minimise opioid-related side effects and enhance recovery [11].

Intraoperative opioids could induce hyperalgesia, wich increase pain intensity and opioid consumption. While intraoperative DEX infusion may be a new and effective treatment option for preventing opioid-induced hyperalgesia [12].

Study of Volkov et al. [13] reported that dexmedetomidine led to a decreased requirement for opioid analgesics, inhaled anesthetics, and the incidence of severe circulation problems during traumatic phases of surgeries. Premedication with a single intravenous dose of 0.5 μg/kg dexmedetomidine decreased the intraoperative propofol and postoperative analgesic requirements, and increased the postoperative satisfaction and sedation level considerably in patients undergoing laryngoscopic biopsy under total intravenous anesthesia [14]. A randomized, double-blind, multicenter study reported that dexmedetomidine is an effective anesthetic adjuvant for patients undergoing local anesthesia for a broad range of surgical procedures, providing better patient satisfaction, lower opioid requirements, and less respiratory depression than placebo [15].

In this randomized controlled study we have similar results, in patients undergoing elective LC, − DEX infusion was associated with a marked decrease in postoperative morphine consumption, lower incidence of severe postoperative pain, and significantly longer time to first use of rescue analgesia. However, the median pain intensity measured with a VRS did not differ between the groups 3, 6, 12 or 24 h after surgery. Other studies also shoved synergic action of intraoperative dexmedetomidine with local anesthetics on postoperative acute pain after craniotomy [16], morphine-sparing effect and significantly lower pain intencity after hysterectomy [9]. Meta-analysis also shoved that opioid-PCA strategies decrease postoperative pain intensity and opioid consumption [17].

Regarding adverse events in this study, there was no inter-group difference in the incidence of postoperative sedation (*p* = 0.7). Other studies showed a higher incidence of sedation in the DEX group, with a mean difference on the Ramsay Sedation Scale of 1.60 units (95% CI 1.49–1.71 units) [18, 19]. Incidence of postoperative nausea and vomiting was reduced in Group D (OR 5, 95% CI 1.1–26, *p* = 0.005), which is similar with what was observed in another study [6] and meta-analysis [17]. In our study DEX didn’t significantly influence the incidence of pruritus, however in meta-analysis it did [17]. Concerning the incidences of hypotension and bradycardia, no differences were found in this study; the incidence of hypertension was significantly higher in Group C (OR 13.8, 95%CI 4–48, *p* < 0.0001). Similar results were reported by other authors [8, 9], with tachycardia and hypertension being registered in seven (35%) and six patients (30%) in the control group, compared with one (5%) and two (10%) patients in the DEX group. In an analysis of 364 patients from seven intermediate- to high-quality randomized controlled trials, it was found that dexmedetomidine was associated with a 3.7-fold increase in transient reversible bradycardia [20]. Other autors also report common adverse events associated with dexmedetomidine, such as bradycardia and hypotension, are predominately mild to moderate in severity [18].

The limitations of this study include the partially blinded design and the small sample size (*n* = 70).

Our trial supports the use of i.v. DEX as an analgesic adjuvant for LC and provides efficacy and safety data. In the authors’ opinion, we have enough data to consider DEX as a valid adjuvant to intraoperative opioids during elective LC.

## Conclusions

Intraoperative DEX infusion is a safe and effective method for improving analgesia during elective LC. DEX appears to significantly reduce the number of patients with severe postoperative pain, postoperative morphine consumption and prolong time to first use of rescue analgesia, while the incidence of postoperative nausea/vomiting or cardiovascular events is similar to or less than with saline control.

## Abbreviations

ANI: Analgesia nociception index
AP: Arterial pressure
ASA: American Society of Anesthesiology
BIS: Bispectral index
CI: Confidence interval
DEX: Dexmedetomidine
ECG: Electrocardiogram
i.v.: intravenous
ICU: Intensive care unit
IQR: Interquartile range
LC: Laparoscopic cholecystectomy
OR: Odds ratio
RASS: Richmond Agitation-Sedation Scale
VRS: Verbal rating scale
